# Development of fluorescent *Escherichia coli* for a whole-cell sensor of 2ʹ-fucosyllactose

**DOI:** 10.1038/s41598-020-67359-x

**Published:** 2020-06-29

**Authors:** Jonghyeok Shin, Myungseo Park, Chakhee Kim, Hooyeon Kim, Yunjeong Park, Choongjin Ban, Jong-Won Yoon, Chul-Soo Shin, Jae Won Lee, Yong-Su Jin, Yong-Cheol Park, Won-Ki Min, Dae-Hyuk Kweon

**Affiliations:** 1 Department of Integrative Biotechnology, College of Biotechnology and Bioengineering, Sungkyunkwan University, Suwon, 16419 Republic of Korea; 2 Carl R. Woese Institute for Genomic Biology, University of Illinois at Urbana-Champaign, Urbana, IL 61801 USA; 3 Biomedical Institute for Convergence, Sungkyunkwan University, Suwon, 16419 Republic of Korea; 4 AP Technology, Suwon, 16229 Republic of Korea; 5 Department of Food Science and Human Nutrition, University of Illinois At Urbana−Champaign, Urbana, IL 61801 USA; 6 Department of Bio and Fermentation Convergence Technology, Kookmin University, Seoul, 02707 Republic of Korea; 7 Department of Food Science and Industry, Kyungil University, Gyeongsan, 38428 Republic of Korea; 8 Center for Biologics, Sungkyunkwan University, Suwon, 16419 Republic of Korea

**Keywords:** Biological techniques, Biotechnology

## Abstract

2′-Fucosyllactose (2′-FL), a major component of fucosylated human milk oligosaccharides, is beneficial to human health in various ways like prebiotic effect, protection from pathogens, anti-inflammatory activity and reduction of the risk of neurodegeneration. Here, a whole-cell fluorescence biosensor for 2′-FL was developed. *Escherichia coli* (*E. coli*) was engineered to catalyse the cleavage of 2′-FL into l-fucose and lactose by constitutively expressing α-l-fucosidase. *Escherichia coli* ∆L YA, in which *lacZ* is deleted and *lacY* is retained, was employed to disable lactose consumption. *E. coli* ∆L YA constitutively co-expressing α-l-fucosidase and a red fluorescence protein (RFP) exhibited increased fluorescence intensity in media containing 2′-FL. However, the presence of 50 g/L lactose reduced the RFP intensity due to lactose-induced cytotoxicity. Preadaptation of bacterial strains to fucose alleviated growth hindrance by lactose and partially recovered the fluorescence intensity. The fluorescence intensity of the cell was linearly proportional to 1–5 g/L 2′-FL. The whole-cell sensor will be versatile in developing a 2′-FL detection system.

## Introduction

Human milk oligosaccharides (HMOs) are present in human breast milk and are closely associated with health benefits. HMOs act as decoys for pathogens (e.g., virus, bacteria, and protozoa) by inhibiting their ability to bind to the surface of epithelial cells^1–4^. Fucosylated oligosaccharides which are not found in bovine milk account for 50% of total HMOs. The HMOs 2′-fucosyllactose (2′-FL; Fuc-α1,2-Gal-β1,4-Glc) and 3-fucosyllactose (3-FL; Gal-β1,4-Fuc-α1,3-Glc), with a concentration range of 0.5–2 g/L, account for the largest portion of fucosylated HMOs^5,6^. Fucosylated HMOs circulate systemically, affecting the host immune response, the regulation of tumour metastasis, and resistance to bacteria, fungi, and other pathogens^2,7,8^. The concentration of 2′-FL in breast milk affects the ability to protect against vital systemic infections in nursing infant^8^. While low content of 2′-FL in breast milk has been associated with a higher rate of diarrhoea during lactation^9,10^, about 20% of human milk do not contain 2′-FL^11^. For these reasons, 2′-FL and 3-FL are spotlighted as nutraceutical and pharmaceutical ingredients; thus, a simple and visually measurable method is indispensable in evaluating their level in breast milk.

Various methods have been developed for the production of 2′-FL, including whole-cell biocatalysis^12–20^, enzymatic synthesis^21,22^, and chemical synthesis^23,24^. Regardless of the method used, a simple detection and quantification method is indispensable for the development of 2′-FL production. Quantification of 2′-FL has been mostly done using high-performance liquid chromatography (HPLC), high-pH anion exchange chromatography, and liquid chromatography–mass spectrometry (LC–MS)^15,25–28^. However, those types of equipment are time-consuming, labour-intensive, and expensive. Therefore, those methods are unlikely to be used in a high-throughput manner or for a brief test.

**Figure 1:**
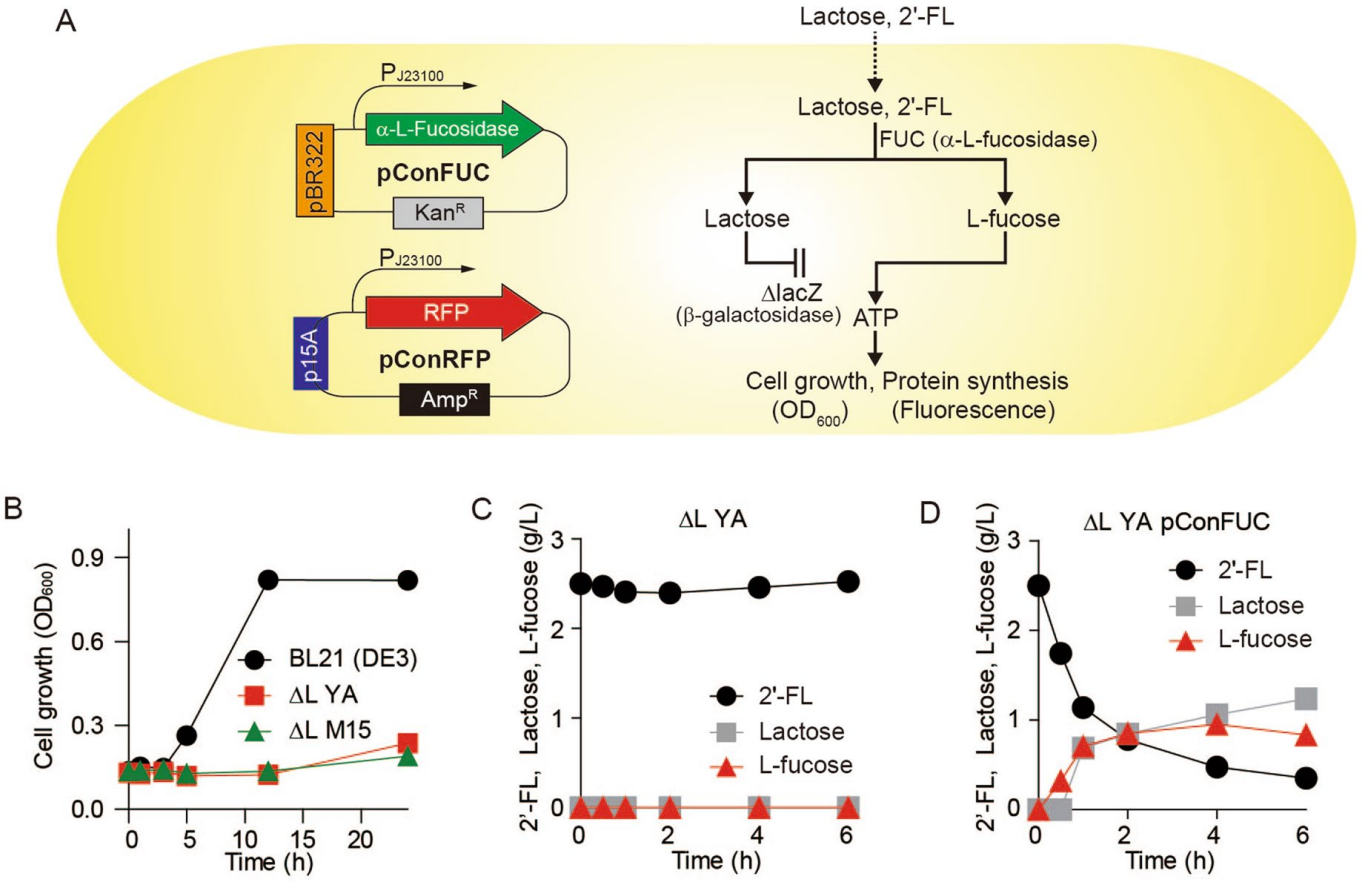
Schematic of a fluorescent *E. coli* 2′-FL biosensor. (**A**) Digestion of 2′-FL by α-l-fucosidase in *E. coli*. 2′-FL is cleaved to L-fucose and lactose by α-l-fucosidase. When *lacZ* is deleted, L-fucose released from 2′-FL is the sole carbon source for cell growth and fluorescence protein expression. Schematic was designed using Adobe Illustrator CS6 (v16.0.0, Adobe Systems Inc., USA). (**B**) Growth of *E. coli *strains using lactose as a carbon source. *E. coli* strains were cultured in R medium, which included 2 g/L lactose. Symbols denote the cell densities of *E. coli* BL21 (DE3) (filled circle), ∆L YA (filled square), and ∆L M15 (filled upward traingle). Cleavage of 2′-FL by soluble lysates of (**C**) *E. coli* ∆L YA and (**D**) *E. coli* ∆L YA pConFUC. Symbols denote the concentrations of 2′-FL (filled circle), Lactose (filled square), and L-fucose (filled upward traingle).

Recently, we developed 2′-FL quantification assays through two-step enzymatic reaction^19^ or one-pot reaction^20^ of fucosidase and fucose dehydrogenase, where 2′-FL concentration is spectroscopically readout by reduction of NADPH. In this study, we developed a visual detection method for 2′-FL using an *E. coli* strain expressing an α-l-fucosidase (FUC, E.C. 3.2.1.63) and a red fluorescence protein (RFP), which can be used in high-throughput applications. l-fucose was released from 2′-FL inside the bacterial cell by α-l-fucosidase, which specifically catalyses the hydrolysis of α1-2-linked l-fucopyranosyl residues from various oligosaccharides^29^. L-fucose was then used for RFP synthesis (Fig. 1A). The *lacZ*-deficient *E. coli* strain was used to disable lactose consumption by cells. This engineered strain semi-quantitatively reported the 2′-FL content in milk in high-throughput by emitting fluorescence in a 2′-FL concentration-dependent manner.

## Results

### Evaluating 2′-FL-dependent cell growth and fluorescence using a whole-cell biosensor

A whole-cell biosensor, which grows and emits fluorescence only in the presence of 2′-FL, was designed (Fig. 1A). The plasmid pConFUC was constructed to constitutively express α-l-fucosidase under the control of the J23100 promoter (Fig. 1A). The 2′-FL can be hydrolysed by α-l-fucosidase in *E. coli* so that the amount of released l-fucose and lactose are proportional to 2′-FL concentration. The cells were engineered not to metabolize lactose by deleting their endogenous β-galactosidase gene (*lacZ*) while retaining *lacY* to import 2′-FL. Two *E. coli* BL21 (DE3) variants, ∆L YA and ∆L M15, with different levels of residual β-galactosidase activity, were cultured in R medium containing 2 g/L lactose^13,16^. Both variants did not grow with lactose as a sole carbon source in a minimal medium for 24 h, whereas wild-type *E. coli* BL21 (DE3) grew well using lactose (Fig. 1B). The difference of growth rate of ∆L M15 and ∆L YA in lactose was marginal. For the strict restriction of lactose consumption, the *E. coli* ∆L YA strain which has much lower β-galactosidase activity was used in the following studies^13,16^.

The active expression of α-l-fucosidase in *E. coli* ∆L YA was examined by analysing the 2′-FL cleavage in the soluble fraction of *E. coli* ∆L YA cell extracts. The cell extracts of wild type *E. coli* BL21 (DE3) and *E. coli* ∆L YA (Fig. S1A and Fig. 1C) did not digest 2′-FL. In contrast, 2′-FL was rapidly digested to release lactose and L-fucose by the cell extracts of *E. coli* BL21 (DE3) pConFUC (Fig. S1B) and *E. coli* ∆L YA pConFUC (Fig. 1D). These results suggest that α-l-fucosidase was actively expressed in *E. coli* regardless of *lacZ* deletion.

### 2′-FL detection by the increase in fluorescence intensity

**Figure 2:**
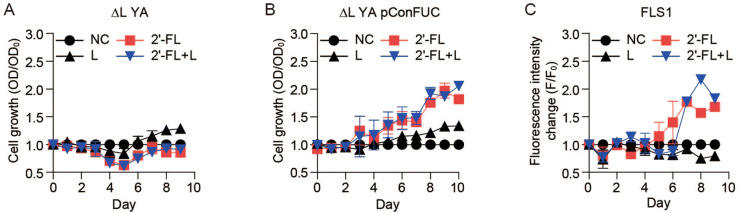
Detection of 2′-FL through RFP fluorescence of *E. coli* strains. *E. coli* ∆L YA, ∆L YA pConFUC, and FLS1 strains were cultured in R medium, which contained lactose, 2′-FL or mixture of lactose and 2′-FL. Relative optical density (ROD) of (**A**) *E. coli* ∆L YA, (**B**) *E. coli* ∆L YA pConFUC, (**C**) Fluorescence intensity change (F/F_0_) of RFP in FLS1 (*E. coli* ∆L YA pConFUC/pConRFP). Symbols denote the negative control (filled circle), 2 g/L 2′-FL (filled square), 2 g/L lactose (filled upward traingle), and 2 g/L lactose + 2 g/L 2′-FL (filled downward triangle). Results are the average of biological replicates (n = 2). Error bars represent standard deviations and are not displayed when smaller than symbol size.

*E. coli* ∆L YA or ∆L YA pConFUC were inoculated with R medium containing no carbon source, 2 g/L 2′-FL, 2 g/L lactose, or a mixture of 2 g/L 2′-FL and 2 g/L lactose. *E. coli* ∆L YA did not grow with any carbon source while *E. coli* ∆L YA pConFUC grew slowly in the presence of 2′-FL (Fig. 2A,B, Table S2) and did not grow using lactose as a sole carbon source. However, lactose did not affect the cell growth of *E. coli* ∆L YA pConFUC using 2′-FL as the carbon source (Fig. 2B).

*E. coli* ∆L YA pConFUC was co-transformed with pConRFP expressing a red fluorescence protein (RFP) under the control of the J23100 promoter. *E. coli* ∆L YA pConFUC/pConRFP (FLS1) cells had a long lag period of 4 days after their inoculation with R medium containing 2 g/L 2′-FL or a mixture of 2′-FL and lactose. Cells did not grow in R medium containing only lactose. RFP signal began to increase on the fifth day of bacterial inoculation in R medium containing 2′-FL (Fig. 2C). Although FLS1 showed a 2′-FL-dependent cell growth and a fluorescence increase, there was a few-days of long lag period. We thought that the cell was not ready to metabolize fucose even after 2′-FL cleavage to fucose and lactose.

### Adaptation of the whole-cell biosensor to L-fucose for faster response toward 2′-FL

**Figure 3:**
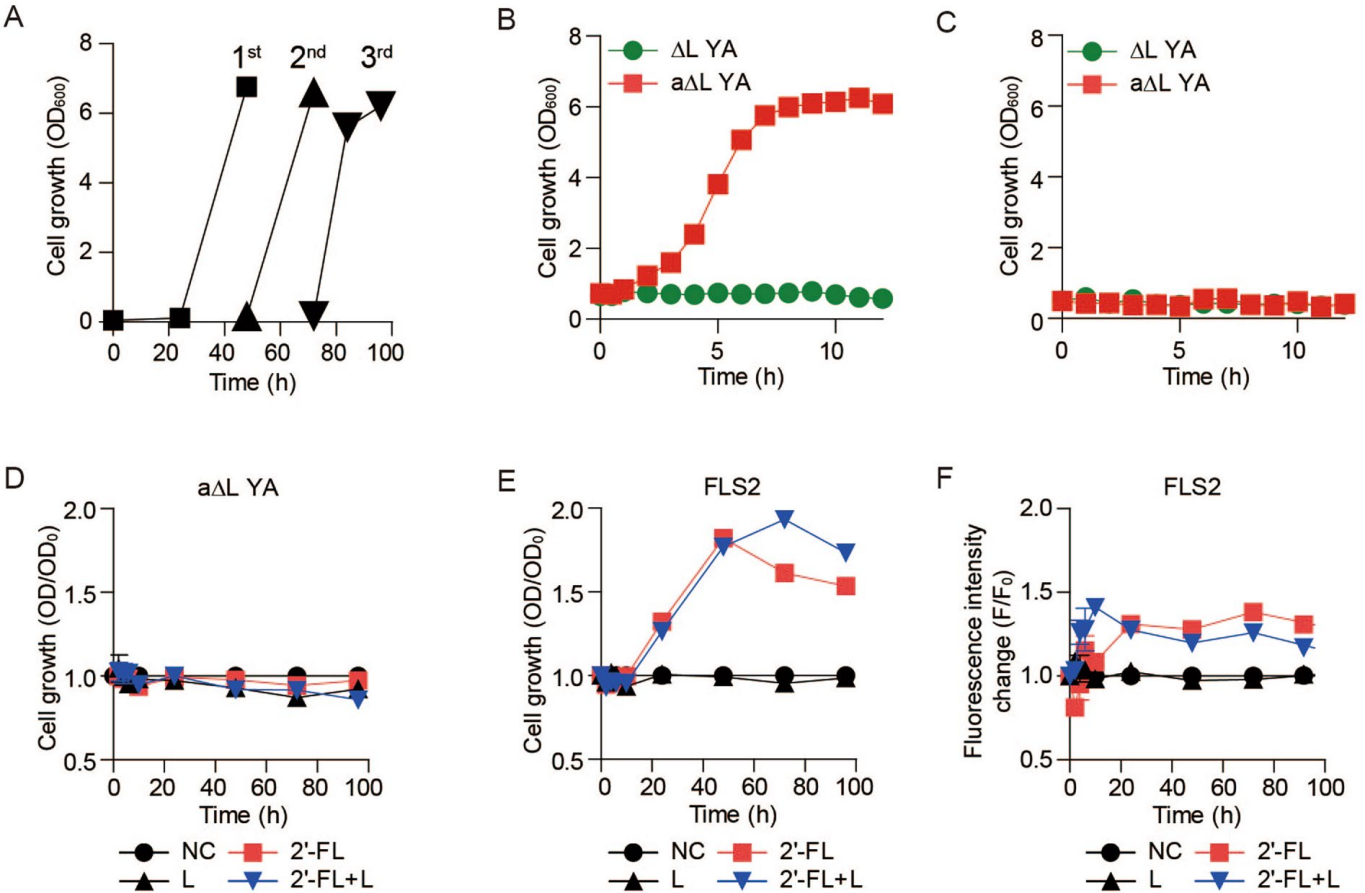
Adaptation of *E. coli* ∆L YA to the L-fucose. (**A**) Repeated culture of *E. coli* ∆L YA for adaptation to L-fucose. (**B**) Growth curves of ∆L YA and adapted ∆L YA (a∆L YA) in R medium containing 10 g/L L-fucose after preculture in L-fucose-containing R medium. ∆L YA (filled circle); a∆L YA (filled square). (**C**) Growth curves of ∆L YA and a∆L YA in R medium containing 10 g/L L-fucose after preculture in LB. ∆L YA (filled circle); a∆L YA (filled square). (**D**) Cell growth of a∆L YA in R medium containing 2′-FL and/or lactose. (**E**) Cell growth of FLS2 in R medium containing 2′-FL and/or lactose. (**F**) Fluorescence intensity change (F/F_0_) of RFP in FLS2 (*E. coli* ∆L YA pConFUC/pConRFP preadapted to L-fucose). Negative control (filled circle), 2 g/L 2′-FL (filled square), 2 g/L lactose (filled upward traingle), and 2 g/L lactose + 2 g/L 2′-FL (filled downward triangle). Results are the average of biological replicates (n = 2). Error bars represent standard deviations and are not displayed when smaller than symbol size (**d–f**).

As the l-fucose metabolic pathway should be activated to use l-fucose as a carbon source^30^, we tested whether the delayed response can be resolved by pre-adapting cells to l-fucose to shorten the lag period. Cells precultured in LB broth showed a long lag period lasting a day in R medium containing L-fucose (Fig. 3A). When *E. coli* ∆L YA was preadapted in R medium containing 10 g/L of L-fucose before inoculation for several rounds, the lag period was dramatically shortened, and the cells immediately entered the exponential growth phase upon inoculation (Fig. 3B). When adapted cells were precultured in the LB broth a second time, cell growth on the L-fucose was retarded similarly to the non-adapted cells (Fig. 3C). These results suggest that this fast-growing phenotype arose because of simple adaptation to the substrate.

*E. coli* ∆L YA pConFUC/pConRFP preadapted to L-fucose (denoted as FLS2) was cultured in R medium containing 2 g/L L-fucose. While *E. coli* ∆L YA lacking the plasmid for fucosidase did not grow in any media for 4 days, FLS2 showed exponential growth after a short lag period (< 8 h; Fig. 3D,E). Notably, the RFP fluorescence reached its maximal fluorescence within 10–20 h when FLS2 was grown in R medium containing 2′-FL (Fig. 3F). The fluorescence emission lasted for 4 days at a stable emission. Cells did not emit fluorescence using lactose as a sole carbon source and 2 g/L lactose present together with 2′-FL did not alter fluorescence intensity either. Although RFP intensity increased by only ~ 50%, these results suggest that RFP fluorescence was a relatively fast and reliable reporter of 2′-FL.

### Enhanced fluorescence intensity

**Figure 4:**
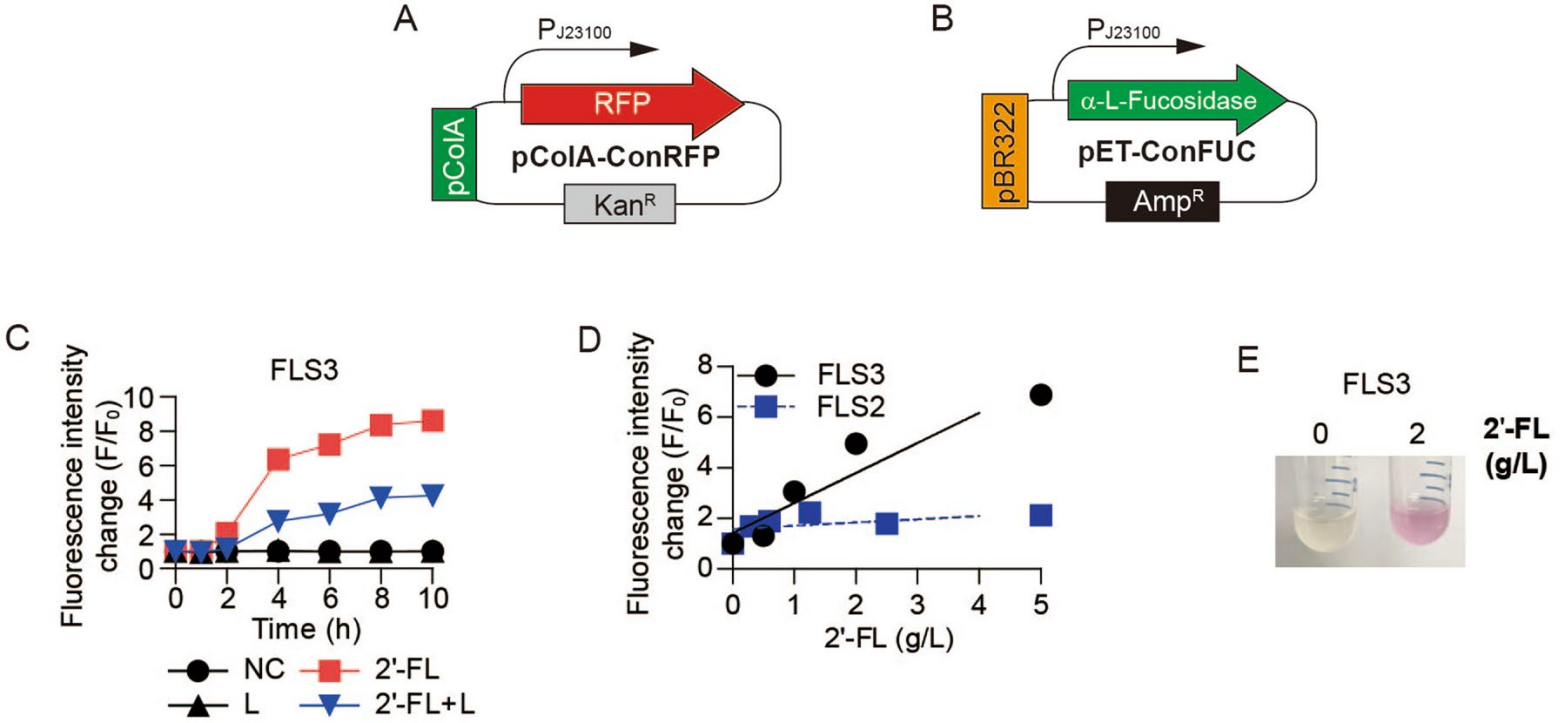
Improved 2′-FL detection. (**A**) Plasmid pColA-ConRFP for enhancing RFP expression. (**B**) Plasmid pET-ConFUC for α-l-fucosidase expression. (**C**) Fluorescence intensity change (F/F_0_) of RFP in FLS3 (*E. coli* ∆L YA pET-ConFUC/pColA-ConRFP). Symbols denote negative control (filled circle), 2 g/L 2′-FL (filled square), 2 g/L lactose (filled upward traingle), and 2 g/L lactose + 2 g/L 2′-FL (filled downward triangle). (**D**) Comparison of FLS2 and FLS3 for 2′-FL-dependent fluorescence response. FLS3, ∆L YA pET-ConFUC/pColA-ConRFP (filled circle); FLS2, a∆L YA pConFUC/pConRFP (filled square). (**C, D**) Results are the average of biological replicates (n = 3). Error bars represent standard deviations and are not displayed when smaller than symbol size. (**E**) A picture of FLS3 cultured in the presence or absence of 2′-FL for 10 h (Left, R media only; Right, R media containing 2 g/L 2′-FL). The representative image from biological replicates (n = 3) was processed using Adobe Illustrator CS6 (v16.0.0, Adobe Systems Inc., USA).

To quicken the 2′-FL detection and increase fluorescence intensity, the expression cassettes of RFP and fucosidase were recombined using different vectors. The small copy number (10–12) replicon p15A was replaced by the high copy number (20–40) replicon ColA for RFP expression (Fig. 4a)^31^. The plasmid pET-ConFUC was also constructed by replacing the kanamycin resistance cassette of pConFUC with the ampicillin resistance cassette of pET (Fig. 4B and Table 1). The *E. coli* ∆L YA strain was then transformed with the resulting plasmids pET-ConFUC and pColA-ConRFP. When *E. coli* ∆L YA pET-ConFUC/pColA-ConRFP (FLS3) was inoculated with R medium containing 2′-FL, fluorescence increased after a short lag phase (< 2 h; Fig. 4C). Furthermore, the maximum fluorescence intensity change was ~ 7 times higher than previous transformants containing pConRFP. The new whole-cell sensor not only exhibited much stronger fluorescence than the previous one the fluorescence intensity change was proportional to a 2′-FL concentration of 1–5 g/L (Fig. 4D and Fig. S2). The signal was strong enough to enable the visualization of the fluorescence (Fig. 4E).

**Table 1:** List of strains and plasmids used in this study.

**Strain/plasmid**	**Relevant description**	**References**
*E. coli* Top10	F–mcrA Δ(mrr-hsdRMS-mcrBC) Φ80lacZΔM15 ΔlacX74 recA1 araD139 Δ(ara leu) 7,697 galU galK rpsL (StrR) endA1 nupG	Invitrogen
∆L YA	BL21star (DE3) ΔL Tn7::lacYA (lacZ deleted)	^13^
∆L M15	BL21star (DE3) ΔlacZYA Tn7::lacZΔM15 (lacZ disrupted)	^16^
FLS1	∆L YA harbouring pConFUC, pConRFP	This study
FLS2	Fucose-adapted FLS1	This study
FLS3	∆L YA harbouring pET-ConFUC, pColA-ConRFP	This study
FLS4	Fucose-adapted FLS3	This study
pConFUC	J23100 constitutive promoter/express α-L-fucosidase/kanamycin resistance/pBR322 replication origin	This study
pET-ConFUC	J23100 constitutive promoter/express α-L-fucosidase/ampicillin resistance/pBR322 replication origin	This study
pConRFP	J23100 constitutive promoter/express RFP/ampicillin resistance/p15A replication origin	This study
pColA-ConRFP	J23100 constitutive promoter/express RFP/kanamycin resistance/pColA replication origin	This study

### Reduced lactose toxicity by fucose adaptation

**Figure 5:**
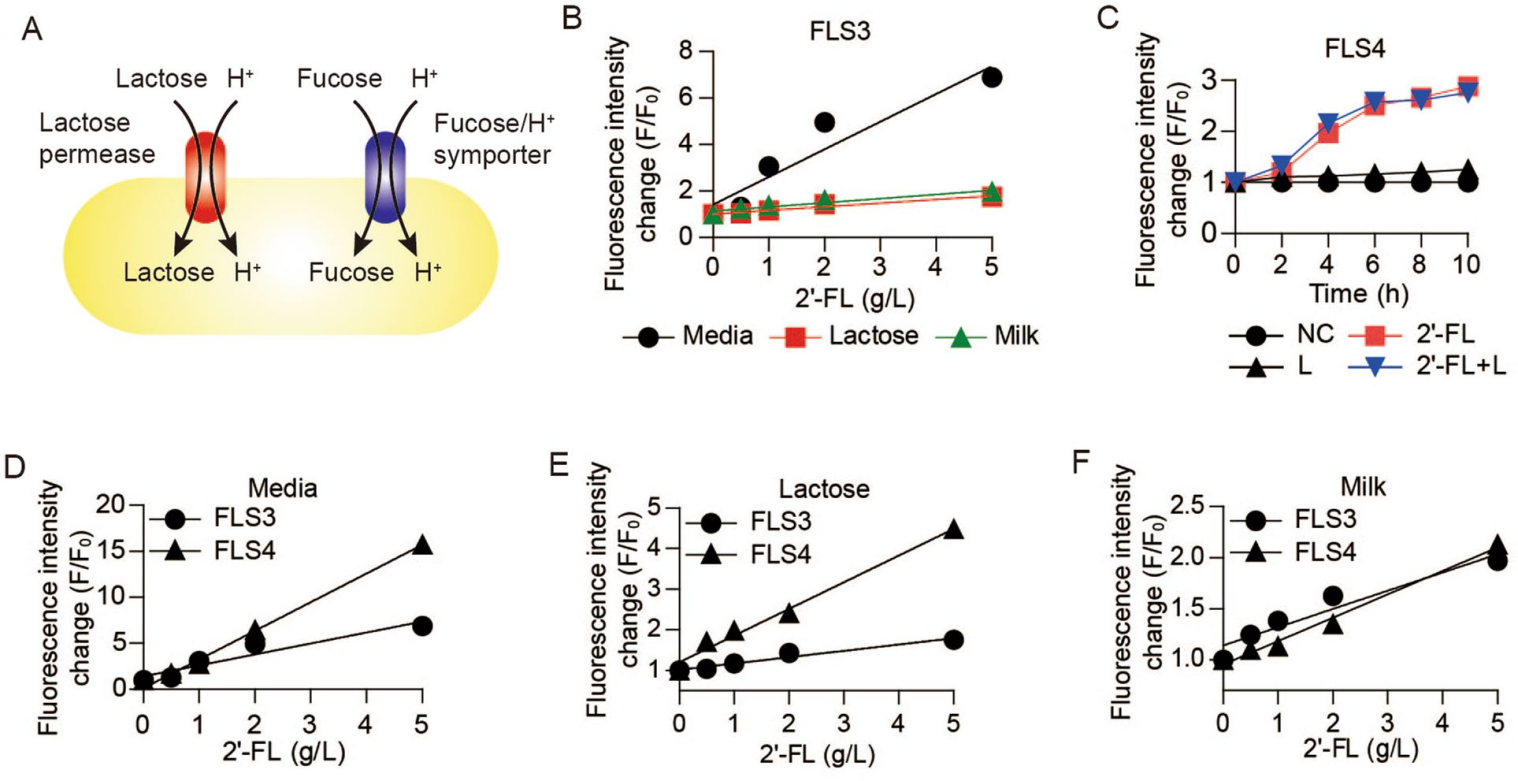
Pre-adaption of the biosensor to fucose to overcome lactose-induced cytotoxicity. (**A**) Acid adaptation with fucose. Lactose permease symports protons as well as lactose lowering intracellular pH. The fucose/H^+^ symporter also symports fucose and proton^33^. Fucose adaptation induces not only a fucose assimilation pathway but also the acid tolerance mechanism. Schematic was designed using Adobe Illustrator CS6 (v16.0.0, Adobe Systems Inc., USA). (**B**) Fluorescence intensity of FLS3 decreased in the presence of lactose. FLS3 (∆L YA pET-ConFUC/pColA-ConRFP) was supplemented with 2 g/L 2′-FL (filled circle), 2 g/L 2′-FL + 50 g/L Lactose (filled square), and 2 g/L 2′-FL + bovine milk (filled upward traingle). Fluorescence intensity was measured after 10 h incubation. (**C**) FLS4 (a∆L YA pET-ConFUC/pColA-ConRFP), which was preadapted to fucose, was cultured in the presence or absence of lactose and 2′-FL. Symbols denote negative controls (filled circle), 2 g/L 2′-FL (filled square), 2 g/L lactose (filled upward traingle), and 2 g/L lactose + 2 g/L 2′-FL (filled downward triangle). (**D–F**) Comparison of FLS3 and FLS4 for 2′-FL-dependent fluorescence response. (**D**) Fluorescence intensity became stronger when the cells were preadapted to fucose. (**E**) FLS4 exhibiting higher fluorescence intensity than the non-adapted cell in the presence of 50 g/L lactose. (**F**) The increase in the fluorescence intensity proportional to 2′-FL when supplemented in bovine milk. Average from biological replicates is shown (n = 3). Error bars represent standard deviations and are not displayed when smaller than symbol size (**B–F**).

We observed that the enhancement of fluorescence intensity by the new combination of expression cassettes was hindered by the presence of only 2 g/L lactose (Fig. 4C). Indeed, media containing 50 g/L lactose or bovine milk supplemented with 2′-FL showed weak fluorescence intensity (Fig. 5B), indicating the hurdles imposed by lactose-induced cytotoxicity.

*E. coli* symports lactose and protons through *lacY*, the lactose permease structural gene, and acidifies the cytoplasm via lactose transport. This acidification causes cytotoxicity followed by the induction of cellular acid shock, resulting in the reduction of proton motive force, intracellular ATP levels, and cell viability^32^. As mentioned above, to accelerate fucose use, preadaptation to fucose was applied a second time to relieve the negative effects of cytoplasmic acidification by lactose. *E. coli* symports L-fucose and H^+^ by a L-fucose/H^+^ symporter, and the internalized cytoplasmic proton would induce *E. coli* to activate its acid resistance system and relieve cytoplasmic acidification^33^. Therefore, preadaptation to fucose can be used not only to activate L-fucose metabolic pathway but also to enhance acid resistance by bacterial cells (Fig. 5A). Indeed, when ∆L YA pET-ConFUC/pColA-ConRFP was preadapted to fucose (denoted as FLS4), 2 g/L lactose did not hinder the 2′-FL detection (Fig. 5C). Furthermore, pre-adaption to fucose enabled much larger fluorescence intensity change than the non-adapted cells, even in the presence of 50 g/L lactose (Fig. 5D,E). Pre-adaption no longer improved the fluorescence intensity when 2′-FL was added to bovine milk. However, the fluorescence intensity was still proportional to 2′-FL concentration (Fig. 5F). Both biosensors were still able to detect the biologically relevant concentration of 2′-FL mixed in bovine milk (Fig. S3).

## Discussion

We designed and developed a functional *E. coli* whole-cell 2′-FL biosensor based on the growth-coupled red fluorescence emission produced after the cleavage of 2′-FL by recombinant α-l-fucosidase, and we demonstrated that the biosensor could quantify 2′-FL. As the working principle of the biosensor, 2′-FL enters *E. coli* ∆L YA perhaps via *lacY* and then is hydrolysed into L-fucose and lactose by the recombinant α-L-fucosidase in the cytosolic space. Because lactose is an abundant disaccharide in milk, it can be used after hydrolysing into glucose and galactose by β-galactosidase in the wild-type *E. coli*. Therefore, endogenous *lacZ* was deleted completely (∆L YA) or partially disrupted (∆L M15). We confirmed that both mutants did not grow in lactose medium at all, and there was no red fluorescence emitted in ∆L YA in the absence of a carbon source (Fig. 1B). Among the *E. coli* mutants, the ∆L YA strain was chosen as the backbone strain for the transformation with the α-L-fucosidase originating from *Xantomonas manhihotis*. The α-L-fucosidase was active in *E. coli* cytoplasm and efficiently cleaved the α (1 → 2) L-fucose branched site in the trisaccharide 2′-FL and hydrolysed it into lactose and L-fucose (Fig. 1D). L-fucose could be consumed, even though small amount, by *E. coli* cells extracts suggesting that L-fucose can be used as a sole carbon source. The expression of recombinant α-l-fucosidase and complete deletion of *lacZ* allowed cell growth in 2′-FL-containing media (Fig. 2B).

After 2′-FL hydrolysis inside the *E. coli* cell, L-fucose, a hexose, is metabolized into dihydroxyacetone phosphate and L-lactaldehyde by the sequential actions of a permease, an isomerase, a kinase, and an aldolase^30^. Aerobically, L-lactaldehyde is oxidized in two steps to pyruvate using NAD-dependent lactaldehyde dehydrogenase and flavin-linked lactate dehydrogenase, and thus channelling all carbons from L-fucose into the central metabolic pathways involved in ATP and amino acid synthesis^30^. Preadaptation to fucose is likely to accelerate cell growth and RFP production because this pathway is activated.

Lactose causes cytotoxicity in several ways. When lactose is transported into a cell that cannot metabolize lactose, the cellular membrane is damaged, and the membrane potential is disrupted by the so-called lactose-killing effect^34^. Lactose can induce cytoplasmic acidification as it is transported into the cytoplasm with a proton through the lactose permease. The resulting acidification of the cytoplasm induces cellular acid shock and reduces proton motive force, intracellular ATP levels, and cell viability^32^. Our results show that cell preadaptation to fucose might allow to overcome lactose acidification. Because fucose is also transported with a proton, it is likely that the acid response system of *E. coli* can be activated during bacteria exposure to fucose^33^*.*

To the best of our knowledge, this is the first demonstration of a simple and easy quantification method of 2′-FL using a whole-cell biosensor (Table S3). Our biosensor might be applicable for high-throughput screening applications using 96- or 384-well microplates. It has the potential to improve process development, colony selection, quality management, 2′-FL kit development and other steps involved in the production 2′-FL.

## Material and methods

### Chemicals and materials

2′-FL was purchased from AP Technology (Suwon, Korea). L-Fucose was purchased from Carbosynth (Compton, Berkshire, UK). Lactose, trace elements for Riesenberg medium (R medium) and antibiotics were purchased from Sigma-Aldrich (St. Louis, MO, USA).

### Strains and plasmids

The list of strains and plasmids used in this study are listed in Table 1. The gene encoding α-l-fucosidase from *Xanthomonas manihotis* was synthesized from IDT (Coralville, IA, USA)^29,35^. The synthesized gene was cloned into the pET28b expression vector (Invitrogen, Carlsbad, CA, USA). Next, T7 promoter of pET28b was substituted with the J23100 promoter (BBa_J23100, https://parts.igem.org/Promoters/Catalog/Anderson) to construct pConFUC. pBbA5aRFP was purchased from Addgene (Addgene_35280)^36^. The lac UV5 promoter of pBbA5aRFP plasmid was also substituted with the J23100 promoter to construct pConRFP that constitutively expressed RFP. To construct pET-ConFUC, the kanamycin resistance gene of pConFUC was substituted for the ampicillin resistance gene amplified from pETduet (Novagen). pColA-ConRFP was constructed by inserting expression regions from pConRFP (Promoter-RBS-CDS) into pColAduet (Novagen).

### Determination of 2′-FL cleavage by α-l-fucosidase

For the 2′-FL cleavage assay, cells harbouring the pConFUC plasmid were cultured in a 1 L baffled flask containing 200 ml of Luria–Bertani (LB) medium (1% tryptone, 0.5% yeast extract, and 1% sodium chloride). When the optical density (OD) at 600 nm reached 0.5, culture was proceeded at 16 °C and 120 rpm for 16 h. Cells were harvested and the pellet was disrupted using an ultrasonic processor (20% amplitude, cycles of 1 s ON and 1 s OFF for 1 m 30 s, on ice). Cell debris and insoluble proteins were removed by centrifugation at 15,000 × *g* for 1 h. The supernatant was collected and concentrated using Centricon 10 (Amicon Co., Beverly, MA, USA). A 5 g/L of concentrated supernatant was mixed with 2′-FL in R medium. The reaction was performed at 37 °C in the shaking incubator. The reaction mixture was centrifuged at 15,000 × *g* for 10 min, and the supernatant was filtered using 0.45 μm syringe filter.

### Detection of 2′-FL from other carbon sources

*E. coli* cells were pre-cultured in LB medium at 37 °C by shaking at 250 rpm for 16 h. Pre-cultured cells were harvested and washed three times with sterilized phosphate-buffered saline (PBS, 137 mM NaCl, 2.7 mM KCl, 10 mM Na_2_SO_4_, 1.8 mM KH_2_PO_4_, pH 7.4). Cells were inoculated into R medium composed of 4 g/L (NH_4_)_2_HPO_4_, 13.5 g/L KH_2_PO_4_, 1.7 g/L citric acid, 1.4 g/L MgSO_4_, and 10 mL/L trace metal solution, i.e., 10 g/L FeSO_4_, 2.25 g/L ZnSO_4_, 1.0 g/L CuSO_4_, 0.5 g/L MnSO_4_, 0.23 g/L Na_2_B_4_O_7_, 2.0 g/L CaCl_2_, and 0.1 g/L (NH_4_)_6_Mo_7_O_24_, containing various carbon sources^37^. OD and fluorescence intensity were measured during time course (Molecular Devices, Sunnyvale, CA, USA).

### Analytical method

Cell concentration was measured by observing the OD at 600 nm. The fluorescence intensity of RFP was measured using the excitation at 584 nm and emission at 615 nm. The OD and fluorescence intensity were measured using the spectrophotometer (Spectramax M2, Union City, CA, USA). Obtained values in the presence of 2′-FL were divided by the values measured in the absence of 2′-FL to calculate relative optical density (ROD) and relative fluorescence unit (RFU). Concentrations of lactose, L-fucose, and 2′-FL after 2′-FL cleavage were measured by using HPLC (Waters Corporation, Milford, MA, USA) equipped with the Rezex ROA-Organic Acid H^+^ column (Phenomenex, Torrance, CA, USA) and a refractive index (RI) detector. A quantity of 0.01 N H_2_SO_4_ was used as the mobile phase at a flow rate of 0.6 ml/min and 50 °C^16^. All experiments were performed in triplicate.

### Statistics

No statistical method was used to determine the sample size in advance. No random method or blind test was used in the experiment and interpretation of results. Numerical analyses were performed using Graph Pad Prism (Graph Pad Software, San Diego, CA, USA).

## Supplementary information

Supplementary information.

## Data Availability

The datasets generated and/or analyzed during the current study are available from the corresponding author on reasonable request.
