# Supplementary material for: Development of fluorescent Escherichia coli for a whole-cell sensor of 2ʹ-fucosyllactose
Source: Sci Rep. 2020 Jun 29;10:10514. doi: 10.1038/s41598-020-67359-x (PMC7324612; doi:10.1038/s41598-020-67359-x)
Supplement: Supplementary file 1 — Supplementary information. [file 41598_2020_67359_MOESM1_ESM.docx]

**Development of fluorescent *Escherichia coli* for a whole-cell sensor of 2'-fucosyllactose**

Jonghyeok Shin^1,2^, Myungseo Park^1^, Chakhee Kim^1^, Hooyeon Kim^1^, Yunjeong Park^1^, Choongjin Ban^1,3^, Jong-Won Yoon^4^, Chul-Soo Shin^4^, Jae Won Lee^2,5^, Yong-Su Jin^2,5^, Yong-Cheol Park^6^, Won-Ki Min^7,*^, Dae-Hyuk Kweon^1,3,8,*^

**Table S1. List of primers used in this study**

| Primer | Sequence (5′🡪 3′) |
| --- | --- |
| ConFUC FW | TTGACGGCTAGCTCAGTCCTAGGTACAGTGCTAGCTTAACTTTAAGAAGGAGATATACATATGGGCACTACTGATTCACGCCAGCTTG |
| ConFUC BW | ATTTCGCGGGATCGAGATCTCGATCCTCTA |
| ConRFP FW | TTGACGGCTAGCTCAGTCCTAGGTACAGTGCTAGCTTAACTTTAAGAAGGAGATATACATATGGCGAGTAGCGAAGACGTTATCAAAGA |
| ConRFP BW | GCCTAAACGATCTCGATCCTCTACG |
| pET-ConFUC I FW | CTCATGAATTAATTCAAATATTAACGTTTACAATTTCTGGCGGCACGATGGCAT |
| pET-ConFUC I BW | AATACAAGGGGTGTTTTCAAATATGTATCCGCTCATGAGACAATAACCCTGATAAATG |
| pET-ConFUC V FW | AACACCCCTTGTATTACTGTTTATGTAAGCAGACAGTTTTATTGTT |
| pET-ConFUC V BW | GAATTAATTCATGAGCGGATACATATTTGAATGTATTTAGAAAAATAAACAAATAGGG |
| pColA-ConRFP I FW | CCGCATAATCGAAATTTGACGGCTAGCTCAGTCCTAGGTACAGT |
| pColA-ConRFP I BW | TCCGCTCACAATTCCTTAAGCACCGGTGGAGTGACGACCTTCA |
| pColA-ConRFP V FW | GGAATTGTGAGCGGATAACAATTCCCCATCTTAGTATATTA |
| pColA-ConRFP V BW | ATTTCGATTATGCGGCCGTGTACAATACGATTACTTTCT |

**Table S2. Growth rate and doubling time of ΔL YA pConFUC grown in 2 g/L 2'-FL.** Each parameter was calculated in the middle of exponential phase.

|  | **ΔL YA**  **with 2 g/L 2'-FL** | **ΔL YA pConFUC with 2 g/L 2'-FL** |
| --- | --- | --- |
| **specific growth rate (h^-1^)** | No apparent growth | 0.0065±0.0007 |
| **doubling time (h)** | Not determined | 107.65±12.31 |

**Table S3. Side-by-side comparison of 2**'-**FL detecting methods**

|  | **Enzymatic method ^1^** | **One-pot analysis ^2^** | **HPLC ^3^** | **Whole-cell Sensor (this study)** |
| --- | --- | --- | --- | --- |
| **Time** | ~70 min/plate | ~60 min/plate | 19 min/sample | ~4 h/plate |
| **Required material and methods** | FUCS, fucose assay kit, Spectrophotometer | Protein expression and purification, Spectrophotometer | HPLC equipped with an appropriate column | Bacterial cell cultivation, Spectrophotometer (Optional) |
| **Accuracy** | Quantitative | Quantitative | Quantitative | Semi-quantitative |
| **Detection range** | 20 mg/mL - 976 mg/mL | 24 mg/mL - 976 mg/mL | 0.2 mg/mL - 12 mg/ml | 0.5 g/L - 5 g/L |
| **Large number of samples** | Applicable | Applicable | Inapplicable | Applicable |


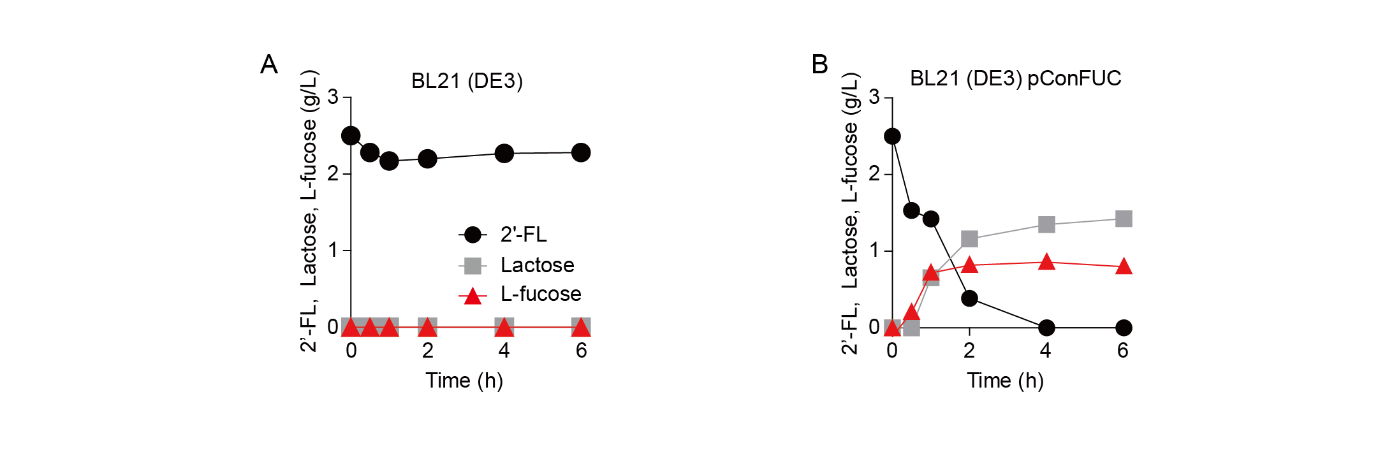
 Supplementary Figure 1. Cleavage of 2'-FL by soluble lysates of (A) *E. coli* BL21 (DE3) and (B) *E. coli* BL21 (DE3) pConFUC.


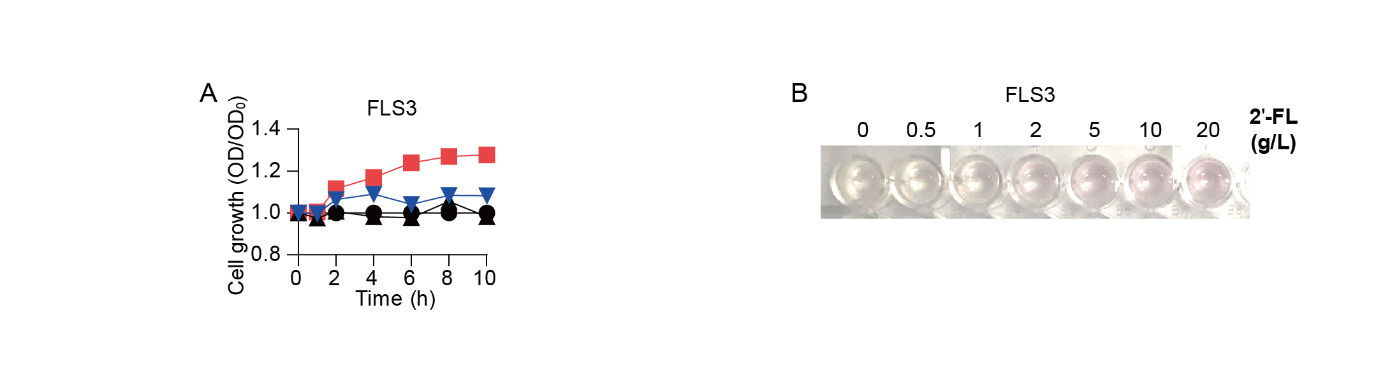
 Supplementary Figure 2. Improvement of 2'-FL detection by enhanced expression of RFP. (A) Cell growth (OD/OD_0_) of FLS3. Results are the average of biological replicates (n=3). Error bars represent standard deviations and are not displayed when smaller than symbol size. (B) Visual detection of 2'-FL with FLS3 after 24 h of incubation. FLS3 was cultured in 2’-FL containing R medium at a final cell concentration of 12.5% in 96-well plate.


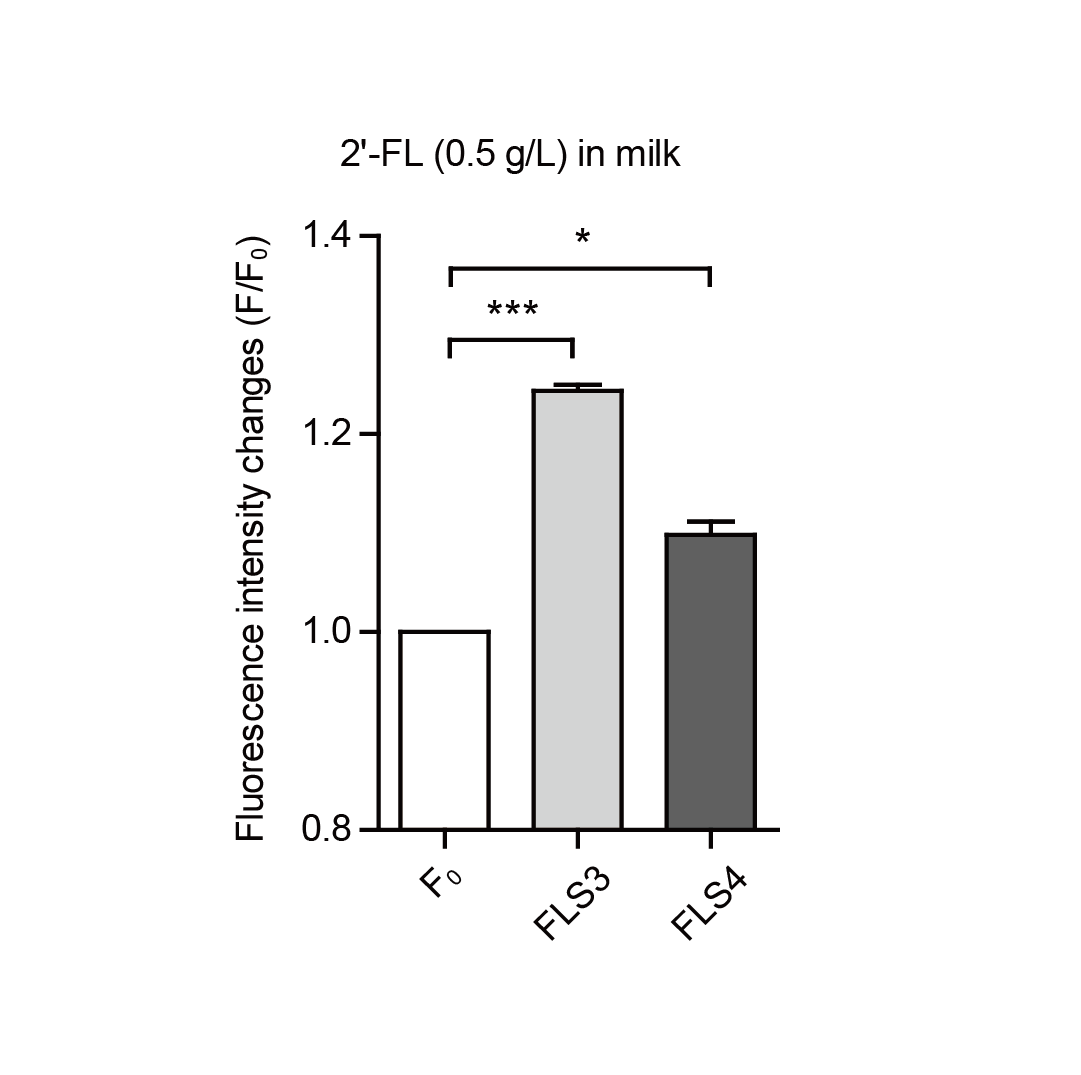


Supplementary figure 3. Detection of biologically relevant concentration of 2'-FL mixed in bovine milk. Fluorescence intensity changes (F/F_0_) of biosensors and the basal fluorescence level (F_0_) were compared after 10-h incubation. Results are the average of biological replicates (n=3). The significance of the experimental results was determined using the student’s t-test (*P < 0.05; **P < 0.01; ***P < 0.001)

**Supplementary References**

1 Seydametova, E., Shin, J., Yu, J. & Kweon, D. H. A simple enzymatic method for quantitation of 2'-fucosyllactose. *J. Microbiol. Biotechn.* **28**, 1141-1146 (2018).

2 Seydametova, E. *et al.* Development of a quantitative assay for 2'-fucosyllactose via one-pot reaction with alpha-1,2-fucosidase and l-fucose dehydrogenase. *Anal. Biochem.* **582**, 113358 (2019).

3 Christensen, A. S., Skov, S. H., Lendal, S. E. & Hornshoj, B. H. Quantifying the human milk oligosaccharides 2'-fucosyllactose and 3-fucosyllactose in different food applications by high-performance liquid chromatography with refractive index detection. *J. Food Sci.* **85**, 332-339 (2020).
